# Analyzing Subway Operation Accidents Causations: Apriori Algorithm and Network Approaches

**DOI:** 10.3390/ijerph20043386

**Published:** 2023-02-15

**Authors:** Yongliang Deng, Ying Zhang, Zhenmin Yuan, Rita Yi Man Li, Tiantian Gu

**Affiliations:** 1 School of Civil Engineering, Suzhou University of Science and Technology, Suzhou 215009, China; 2 School of Mechanics and Civil Engineering, China University of Mining and Technology, Xuzhou 221116, China; 3 School of Management Engineering, Shandong Jianzhu University, Jinan 250101, China; 4 Sustainable Real Estate Research Center, Department of Economics and Finance, Hong Kong Shue Yan University, Hong Kong 999077, China

**Keywords:** subway operation, safety risk, vulnerability evaluation, network theory

## Abstract

Subway operation safety management has become increasingly important due to the severe consequences of accidents and interruptions. As the causative factors and accidents exhibit a complex and dynamic interrelationship, the proposed subway operation accident causation network (SOACN) could represent the actual scenario in a better way. This study used the SOACN to explore subway operation safety risks and provide suggestions for promoting safety management. The SOACN model was built under 13 accident types, 29 causations and their 84 relationships based on the literature review, grounded theory and association rule analysis, respectively. Based on the network theory, topological features were obtained to showcase different roles of an accident or causation in the SOACN, including degree distribution, betweenness centrality, clustering coefficient, network diameter, and average path length. The SOACN exhibits both small-world network and scale-free features, implying that propagation in the SOACN is fast. Vulnerability evaluation was conducted under network efficiency, and its results indicated that safety management should focus more on fire accident and passenger falling off the rail. This study is beneficial for capturing the complex accident safety-risk–causation relationship in subway operations. It offers suggestions regarding safety-related decision optimization and measures for causation reduction and accident control with high efficiency.

## 1. Introduction

**Figure 1:**
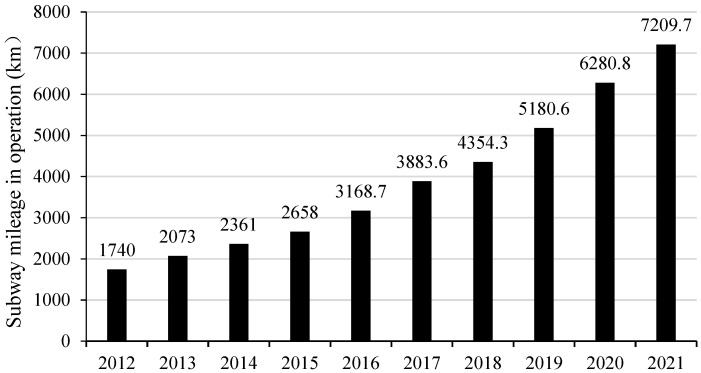
Length of subway lines in China from 2012 to 2021 (Data source: China Urban Rail Transit Association).

With continual urban sprawl, a city’s traffic flow will increase rapidly, and the problem of urban traffic congestion will become more and more serious. Against this backdrop, the subway has quickly developed due to its advantages of high efficiency and punctuality, large transportation volume and low environmental pollution. By the end of 2021, 188 cities in 62 countries and regions had subways which covered 18,952.3 kilometres [1]. China has experienced the most rapid urban rail transit development among these countries. The mileage of subway operations in China is shown in Figure 1. At present, 42 cities in mainland China have opened subway stations, with a total length of 7209.7 kilometres. In addition, the total number of subway passengers in China has reached 16.92448 billion in 2021. Against the backdrop of such a large volume of passengers on subways, it is necessary to accelerate subway construction to ease the road traffic. That said, given the ever-increasing operation lines to satisfy public needs and the complex subway operation network, operational risks are also increasing.

With rapid subway development, subway operation safety has received more attention. Due to the subway system’s complexity and the increasing uncertainty of the external environment, accidents often occur during operations, and the vulnerability of safety risk management in subway operations is increasingly prominent. According to statistics, 1911 subway accidents occurred in 35 cities in mainland China during the 12 years from 2007 to 2018 [2]. Because of the rapid development of subway construction and the high frequency of subway accidents during the operation stage, it is high time to enhance safety management in subway operations. Learning from past accidents is the best approach [3]. Accident analysis has been widely used to improve safety performance in various industries, such as the transport industry [4,5,6], chemical engineering [7] and construction engineering [8].

Many investigations concluded that there might be many precursors or risks without harm and loss before subway operation accidents occur. Based on the general process of accident occurrence and development, various causations frequently happen in the process of subway operation and lead to safety accidents directly or indirectly. According to the theory of track-cross of casualty accidents, accidents are caused by the unsafe status of matters and people’s dangerous behaviours. Aside from accidents, relevant studies should focus on the precursor and risk factors, i.e., conditions, events and sequences that precede and cause an accident. Accident precursor and risk analysis may explore and obtain critical information concerning failure mechanisms and reduce the probability of an accident by reducing corresponding precursors [9,10]. 

Previous studies have explored subway accidents’ causations from the perspective of personnel, equipment, management, environment, etc. Zhang et al. constructed a Shanghai subway operation incident database and analyzed the accident precursors [11]. The results proved that precursor analysis could improve subway operation safety management. Li et al. identified subway operation hazards and analyzed their relationships [12]. However, these studies have not analyzed the correlation between accidents and hazards. Currently, the existing studies have concentrated on a specific city or a particular perspective, mainly focusing on the causes of accidents but ignoring the role of accidents in risk transmission. Accidents may happen due to other accidents. Subway accidents and causal factors must be analyzed simultaneously in an integrated framework.

This study aims to identify the accidents and their causation factors, analyze the relationships between them and their causative factors and control the critical factors for preventing safety accidents and enhancing subway safety performance. This study can help subway operation stakeholders to formulate more effective safety management strategies and practical emergency response plans. For this purpose, an analytical framework is put forward. Then, subway operation accidents are collected, and causative factors are identified through grounded theory. Next, relationships are determined based on association rule analysis. Subsequently, the network model is established and analyzed scientifically. According to the findings and discussions, recommendations are proposed to promote the related research. This study deepens the understanding of subway operation accidents and provides valuable suggestions for subway operations in the long run. 

## 2. Literature Review

The subway is a typical complex infrastructure system with many subsystems. Many of the previous studies have identified subway operation safety risks. Risk identification includes comprehensiveness and systematisms and is mainly carried out by management, equipment and facility, personnel and environment [12]. It should be noted that risk identification must be combined with the characteristics of the subway operation process and characteristics [11]. Kyriakidis et al. analyzed precursors, top events, injuries and deaths and their interrelationships with incidents and accidents in global subways via the maturity model [13]. The results suggested that effective measures could be taken to avoid accidents, according to the analysis of the precursors of the subway system. With the deepening of the research, some new perspectives and methods have been applied. Deng et al. identified critical and vulnerable functional modules in subway equipment and discovered the most dangerous failure mode [14]. Zhang et al. identified and analyzed four fire scenarios in a subway station located in the Wuhan Metro System in China [15]. Forero-Ortiz and Martinez-Gomariz summarised the potential hazards and identified a knowledge map about the impact of water on the subway network [16]. 

Given the diverse, dynamic and complex features of the safety risks in subway operation, many risk analysis and evaluation studies have been carried out based on risk identification. These evaluation methods are mainly divided into qualitative evaluation and quantitative evaluation. Wang et al. employed the grey incidence method to evaluate the hazards of subway dynamic operating systems and conduct a quantitative analysis of operational risks [17]. Fire occupies the highest percentage of frequency and does tremendous damage to subway operations. Roshan evaluated the fire risk of the Tehran metro and estimated its economic loss based on event tree analysis [18]. Avci and Ozbulut presented the threat and vulnerability risk assessment (TVRA) procedure and provided mitigation strategies [19]. As flooding often cause severe damage to the subway, Lyu et al. proposed a perspective method for flood assessment of the subway system [20]. As emergency evacuation in disaster is of great significance to reduce losses, Chen et al. developed a four-dimension parameter system to assess evacuation performance in the subway station [21]. These evaluations deepen the understanding of the safety risks of subway operations. Risk evaluation can provide a basis for effectively avoiding, preventing and controlling the safety risks that may arise in the process of subway operations [22].

Risk management has played a central role in the safe management of subway operations over the years. Since the exposure of staff and passengers to hazards cannot be avoided entirely, risks cannot be eliminated but can be controlled at acceptable levels. Many scholars have studied the safety management of subway operations from different perspectives with proper methods. Xiahou et al. explored the impact of design for safety on subway lifecycle safety management [23]. Di Graziano et al. introduced a risk management methodology which can analyze the causes and consequences and assess the influence factors of subway safety [24]. Kim et al. explored the effects of the built environment in subway stations on pedestrian injuries [25]. In addition, the development of information technology provides a powerful tool for subway safety management, such as the internet of things, the building information model and big data. Kaewunruen et al. introduced a digital twin to evaluate and manage a subway station in Hefei City [26]. Tang et al. used a building information model to reduce the emergency evacuation risk in subway operations [27]. More sophisticated safety management methodologies and tools are crucial in improving the scientific level of management and decision-making.

The existing studies mainly focus on cause analysis, safety risk identification, risk assessment and management. These studies have provided a valuable reference for improving safety in subway operations. However, as causative factors and accidents do not exist in isolation, and further study should be undertaken to explore intra-relationships between accidents, intra-relationships between causative factors and interrelationships between accidents and causative factors in the subway operation process. The relationship between various factors exhibits complexity and dynamics, and the proposed SOACN better represents actual scenarios. Exploring the risk transmission process from a complex network perspective is closer to reality and thus worth further study. Furthermore, causative factors and accidents should be integrated into risk chains or network models. The grounded theory is applicable to determine the risk factors, and associate rule analysis is suitable for obtaining the relationships. The network model can be established and analyzed based on network theory. These three methods are suitable for research work in combination. Therefore, this study proposes an integrated analytical framework to identify and analyze three relationships between accidents and causations based on the Apriori algorithm and network theory. 

## 3. Research Method

### 3.1. The Analytical Framework

**Figure 2:**
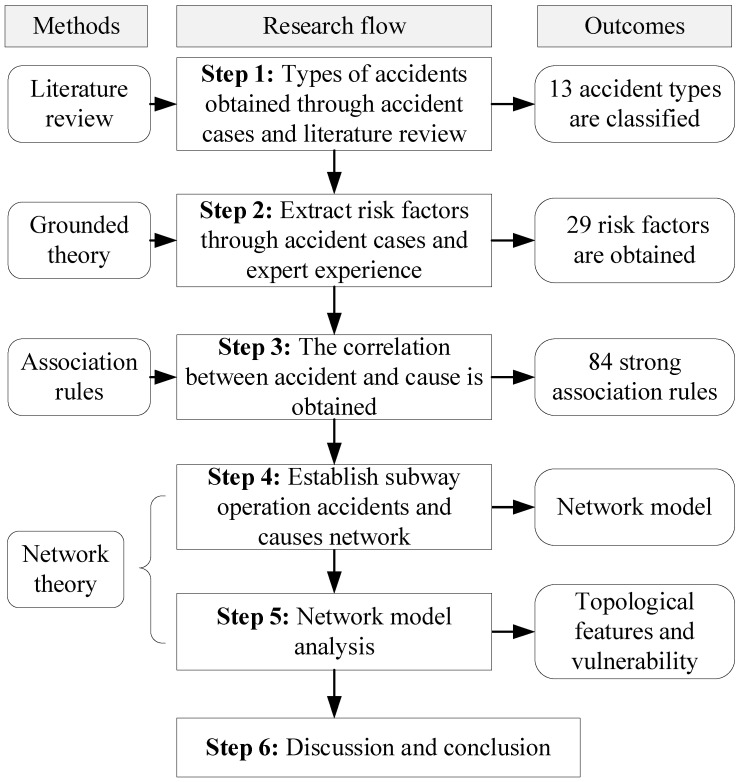
The analytical framework.

Based on this literature review, the leading research status and trends of subway safety management are displayed, and the framework of this research is shown in Figure 2. The overall framework of this research is mainly divided into five steps. The first step is to classify subway accidents during operation into 13 types. The second step is to obtain the causative factors through reviewing the accident cases and expert experience. The third step is to apply the association rule Apriori algorithm for mining potential associations between causative factors and accidents. The fourth step is to employ complex network theory to build the subway operation accident causation network (SOACN). The fifth step is to analyze the topological features and vulnerability of SOACN. Finally, this paper summarises the research results and suggests promoting subway operation safety.

The application of grounded theory in this study can be used to extract causal factors from the collected data and carry out specific classifications. The saturation test ensures the integrity of the constructed causal factors. In general, lacking basic data usually impedes the smooth implementation of network model analysis. Data mining is the task of finding useful information in large datasets. It is believed that the reasonable choice is to use data mining to discover connotative and unknown knowledge [28]. The advantage of employing association rule analysis is its ability to identify association rules for exploring potential relationships. It is an essential link in the field of safety risk management to dig out the possible correlation between accident causes and analyze the characteristics of risk transmission in subway operations.

Nevertheless, it is worth noting that data mining requires raw data or information [29]. Based on reliable data acquisition and processing methods, complex network theory has been recognized as the most appropriate approach to explore the behaviours of dynamic processes occurring on networks. The advantages of network analysis include two aspects. First, this method can build a network model consisting of different causative factors, accidents, and interactions. Second, the risk transmission path can be visualized, and the network topology and dynamic characteristics can be quantitatively calculated and analyzed in-depth.

### 3.2. Grounded Theory

Grounded theory is a qualitative and inductive method in social science, which was first put forward by Glaser and Strauss [30]. It is usually used to establish a theory based on data collection and analysis [31]. Grounded theory emphasizes the generation of a pragmatic theory grounded in the data of experience and text, which has resulted in a profound and enduring impact on qualitative research. Its problem-solving approaches are prerequisites for advanced study in many subject areas. Grounded theory has been widely accepted and applied in a variety of research areas since it was proposed, such as information systems [32], construction management [33,34], and the banking industry [35]. Exploring safety knowledge in subway operation risk management using grounded theory could be a suitable approach. 

**Figure 3:**
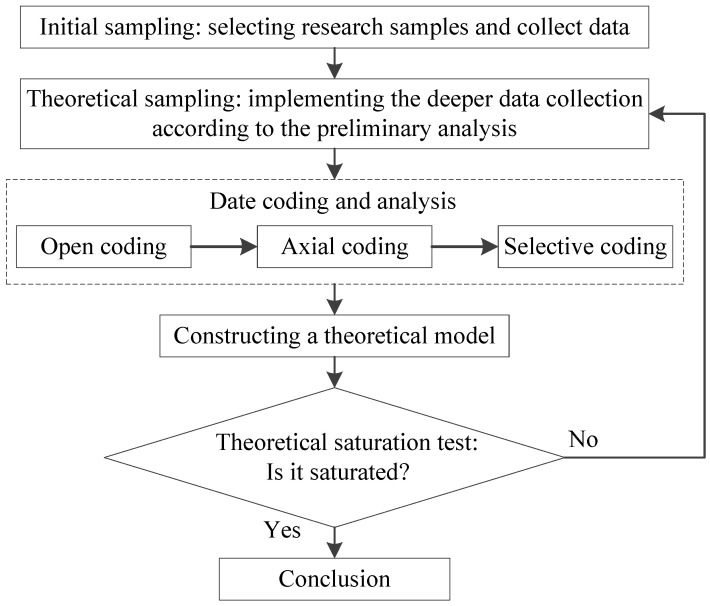
The implementation steps for grounded theory.

The primary process of implementing grounded theory is shown in Figure 3. The research data should be collected first. Secondly, open coding, axial coding, selective coding and theoretical saturation tests are carried out to analyze sequences. In this step, coding refers to the continuous comparison between concepts and events to facilitate the conceptualization of data. Theoretical saturation refers to saturated data and information extracted from the sample. Once the saturation test is completed and verified, the theory will not be affected by the new sample.

### 3.3. Association Rule Analysis

Association rules can be used to mine the relationship between data item sets by calculating the support and confidence of data item sets. The association rule is an essential data mining technology that can explore the potential association and mutual relationship between data item sets [36]. It has been widely recognized and applied in a variety of research areas since it was proposed, including web data analysis [37], recommender systems [38], and disease diagnosis [39]. 

In this field, the Apriori algorithm is the most classic, and some algorithms are developed based on its improvement [40]. The core idea of the Apriori algorithm is to screen all the association rules that satisfy the support and confidence thresholds. It retrieves frequent items through multiple iterative operations, and all frequent items can be calculated through k iterations. Given the collected subway operation accidents, it can obtain valuable strong association rules in accident information.

### 3.4. Network Modelling and Analysis

Complex network theory is a powerful approach to exploring complex systems, such as supply chains [41], decentralized energy systems [42], urban traffic [43,44], and nuclear reactors [45]. The two essential elements in the network model include vertexes and edges that can be abstracted from the research object. The topological properties mainly include degree distribution, betweenness centrality, clustering coefficients, network diameter and average path length, small-world properties and scale-free properties. Vulnerability is a global system characteristic that expresses the magnitude of severe consequences following a specific hazardous event [46]. In a network, this can be reflected by network efficiency. The network efficiency *E* is obtained by Equation (1).

(1) $$E=\frac{1}{n(n-1)}\sum_{i\neq j}\frac{1}{d_{ij}}$$

where *n* is the number of vertexes in the network, and *d_ij_* is the distance between two vertexes.

## 4. Analysis and Results

### 4.1. Data Collection

To collect data on subway operation accidents, terms such as “subway/metro/underground”, “operation” and “accident/incident” were employed to search for accident cases on the internet, including the Chinese National Knowledge Infrastructure (CNKI), Google Scholar and various media websites. A greater quantity of documents, reports and webpages were retrieved and scrutinized. In the meantime, several pertinent texts were chosen as sources of information. Finally, a total of 683 subway operation accident cases were initially collected in this study. After screening the cases that had unclear accident contents and causes, a database of 608 accident cases was finally formed.

By referring to the “Standard of the operation safety assessment for existing metro, GB/T 50438-2007” and the classification of the types of subway operation accidents in the relevant literature [11], this study classified 13 types of subway operation accidents. It includes seven train door/screen door clamping accidents, 37 fire accidents, 23 explosion accidents, 37 poisoning and suffocation accidents, 29 passenger-falling-onto-rails accidents, 10 passenger-falling injury accidents, three stampede accidents, 10 train collision accidents, 17 train rear-end accidents, 17 train derailment accidents, 21 train-hit-people accidents, 10 station/line flooding accidents and 387 operation delay accidents (this type of accident refers to an accident causing operation delay in addition to other accident types listed). Using A as the code of the accident type, this study sets the codes of the 13 accident types as A1 to A13 in sequence. The codes corresponding to specific accident types are shown in Table 1.

### 4.2. Construction of Risk Factor Index System

Grounded theory is employed to find out the core concepts of the collected data and build relevant social theories through the correlation between concepts to build substantive theories from the bottom up. This study randomly selected 4/5 (487) accident cases in the collected subway operation accident cases for grounded coding. The remaining 1/5 (121) accident cases were used to verify whether the grounded coding reached saturation to obtain the causative factor system of subway operation accidents. To avoid the influence of subjectivity, this study combines personal coding with expert experience to improve the rationality and accuracy of coding results. According to the open coding of 487 subway operation accidents, 29 causative factor index subcategories are obtained. The specific process is illustrated in Table 2.

To explore the relationships between subcategories of accident causations, this study finally divides these subcategories into four categories: human factor, mechanical factor, environmental factor, and management factor. The results of spindle coding are listed in Table 3.

Due to the limitations of case-data collection, a theoretical saturation test is needed. The reserved 1/5 (121) accident cases were recoded in this study. The results show that the subcategories and main categories of the spindle coding did not change, and the main categories did not generate new causative factors in the theoretical testing. Therefore, the spindle coding results were determined as the causative factor system of subway operation safety accidents.

### 4.3. Correlation Analysis

This study analyzed the relationships between various accidents and causative factors and explored the potential laws for reducing subway operation safety accidents. The Apriori algorithm was used to mine association rules. Among the 608 accident cases, operation delay accounted for about 72% of total accident cases, while stampede accidents accounted for only 0.5%. Owing to the significant difference in the number of cases of different accident types, the accident type with the smallest proportion is used as the base value to ensure that the obtained correlation is more comprehensive. This study set the frequent item with accidents and causes accounting as 20%, and the rule support is 50%. The accident type with the minimum proportion accounts for 0.5% of total accidents. Furthermore, the minimum support of the rule is 0.1%, and the minimum confidence is 0.5%. After analysis by SPSS software, 82 strong association rules are obtained, as shown in Table 4.

### 4.4. Network Modelling

**Figure 4:**
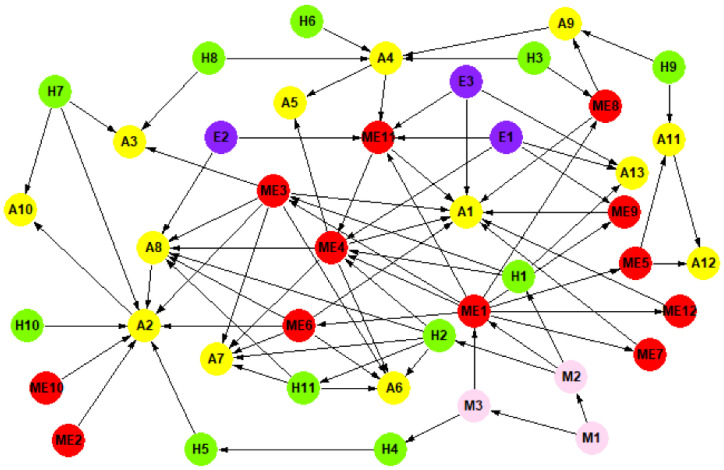
Subway operation accidents causation network (refer to Table 1 and Table 3 for the meaning of the symbols).

Pajek software was selected to visualize the network, as shown in Figure 4. The 13 accident types (Table 1), 29 causative factors (Table 3) and 82 relationships between accidents and causative factors were used to build the SOACN model. The specific accidents and causation factors were regarded as vertexes of the network, and the edge of the network represented the relationships between accidents and causative factors. Therefore, the SOACN included 42 vertexes and 82 directed edges.

### 4.5. Topological Features

Topological features can be used to do statistical analysis of the association relationships between nodes from both local and global perspectives. It helps to understand the SOACN in depth, especially in the exploration of the critical nodes and paths in the network. In this study, the degree distribution, betweenness centrality, clustering coefficient, network diameter, average path length, small-world property and scale-free property have been analyzed as follows.

#### 4.5.1. Degree Distribution

**Figure 5:**
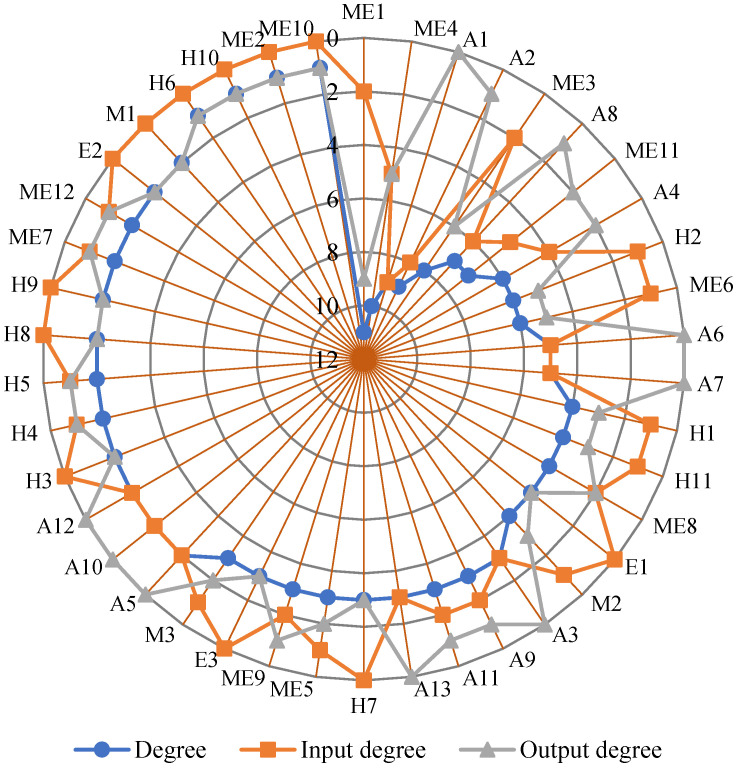
Degree distribution.

In the SOACN, the input degree refers to the total number of adjacent superior vertexes that can transmit the safety risk to this vertex. The output degree refers to the total number of adjacent subordinate vertexes to which that vertex can transmit the risk. The total degree is the sum of the output degree and the input degree. The degree distribution of all vertexes in SOACN is shown in Figure 5. The degree of most vertexes in the network is between 1 and 11.

In contrast, the degree of improper operation and maintenance of equipment and facilities (ME1), signal failure (ME4), operation delay accidents (A1) and fire accidents (A2) are significantly higher, at 11, 10, 9 and 9 respectively. The input degree of operation delay accidents (A1) and fire accidents (A2) are significantly larger than that of other vertexes. The output degree of improper operation and maintenance of equipment and facilities (ME1) is significantly larger than that of other vertexes. The higher the degree of vertexes, the more likely the risks represented by these vertexes will occur together with other risks. Therefore, strengthening the control of these vertexes can better reduce safety risks.

Table 5 shows the average degrees of various types of vertex sets in SOACN. The causation vertex set had a lower average degree than the accident vertex set, which signifies that causation vertexes have fewer neighbour vertexes than accident vertexes. The average input degree of the accident vertex set is much larger than the average output degree of the accident vertex set. It is more significant than the average input degree of the accident vertex set. The average output degree of the causation vertex set is much larger than the average input degree of the causation vertex set. It is larger than the average output degree of the accident vertex set. It denotes that causation vertexes are more critical in amplifying the cascading effects. In reality, accidents are paid more attention because of the serious consequences of economic losses and casualties. However, subway operation safety managers must transform conventional attention and focus more on controlling accidents by reducing their connections with various causation vertexes in the SOACN. In addition, the mechanical factor has the highest average degree, which indicates that the mechanical factor plays a more significant role in the cause of subway operation accidents.

#### 4.5.2. Betweenness Centrality

**Figure 6:**
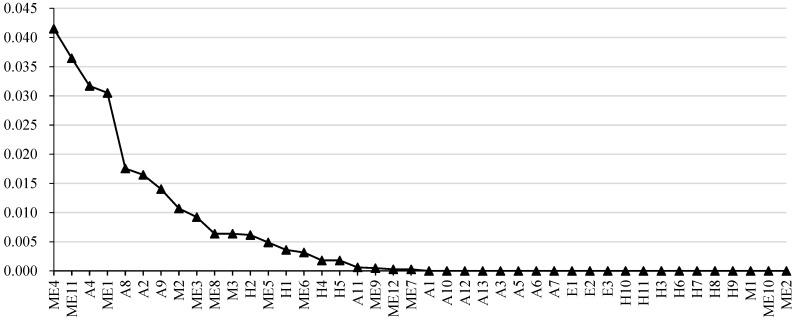
Values of vertex betweenness (see Table 1 and Table 3 for the meanings of abbreviations).

Vertex betweenness is used to describe the extent to which a vertex plays an intermediary role in the interaction between all possible pairs of vertexes in a network [47]. Previous research has studied the vertex betweenness in occupational French and English tweets [48], construction safety videos on YouTube [49] and Twitter knowledge-sharing networks [50]. Yet, none of these threw light on subway accidents. This study showed that the average betweenness of the network is 0.0366, and the betweenness of each vertex is illustrated in Figure 6. The betweenness of most vertexes is less than 0.02.

In contrast, the betweenness of signal failure (ME4), power failure/power supply interruption/power supply device failure (ME11), passenger falling off rails (A4) and improper operation and maintenance of equipment and facilities (ME1) are significantly higher than that of other vertexes. It is not difficult to find that vertexes with large betweenness are mostly mechanical factors, so daily maintenance of equipment should be strengthened to prevent the propagation of risk chains. The vertexes with high betweenness facilitate the transmission efficiency of safety risks higher. Therefore, effectively controlling these vertexes and reducing the possibility of their occurrence will significantly prevent the risk from spreading.

As shown in Table 6, the accident vertex set gains a more considerable value of average betweenness centrality than that of causation 1, causation 3 and causation 4, but less than that of causation 2. This can be explained by mechanical factor vertexes appearing more in shortest paths in the SOACN. In contrast to the human, environmental and management factors, the mechanical factor plays a more intermediary role. This signifies that this factor has a more significant influence under the control of the other factors over safety-risk propagation. The big difference among different vertex sets further demonstrates that it is reasonable to allocate security resources based on causative factor characteristics.

#### 4.5.3. Clustering Coefficient

**Figure 7:**
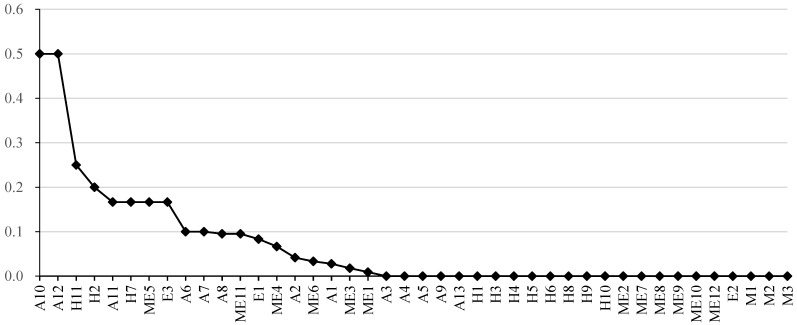
Values of clustering coefficients.

The clustering coefficient of a vertex is defined as the ratio of the actual number of edges to the total number of potential edges between neighbours. The clustering coefficient of each vertex in SAVN is illustrated in Figure 7. The clustering coefficients of the four vertexes in the network are considerably more significant than those of other vertexes in the network, including passenger-falling injuries (A12), poisoning and suffocation accidents (A10), speeding (H11), and driver’s illegal operation (H2). These four vertexes are more closely related to their neighbour vertexes. When these four risks occur, they are likely to be accompanied by correlated risks. The two vertexes with the most significant clustering coefficient are accident vertexes, which shows that these two types of accident vertexes have a high degree of aggregation with the surrounding vertexes. Effectively preventing these two types of accidents can improve safety management performance. The two causative factors with a significant clustering coefficient are human factors, indicating that it is essential to carry out safety education and training. Safety managers should improve workers’ safety awareness and ability, especially for drivers.

As shown in Table 7, the accident vertex set has a more significant value of average clustering coefficient than that of each causation vertex set in SOACN. Compared with causation vertexes, the neighbour vertexes of an accident vertex are more prone to connect. It may be explained by the fact that an accident does not happen alone. On the other hand, the discrepancy among the four types of causation vertex sets is apparent. The average clustering coefficient of causation 4 is 0, indicating no connections between the neighbour vertexes of causation 4.

#### 4.5.4. Network Diameter and Average Path Length

The network diameter of the SOACN is 7. The diameter path is as follows: Passenger congestion (H9) → Train door/screen door clamping accidents (A9) → Passenger falling off rails (A4) → Power failure/power supply interruption/power supply device failure (ME11) → Signal failure (ME4) → Train derailment accidents (A8) → Fire accidents (A2) → Poisoning and suffocation accidents (A10). The path from passenger congestion to poisoning and suffocation accidents has the most vertexes, indicating an indirect correlation. It is difficult for the former to lead to the latter’s occurrence directly, but through the transmission of risks, it may eventually lead to the latter’s occurrence. This study helps to discover potential causal associations that are not obvious. In addition, the average path length of the SOACN is 2.4134, implying that one risk in the network only needs two to three steps on average to reach another risk.

#### 4.5.5. The Small-World Property

The average path length of the SOACN is 2.4134, and the average clustering coefficient of the whole network is 0.0559. This study used Pajek software to randomly simulate 10 networks of the same size (the number of vertexes and edges are the same). The average path length of these 10 random networks is 4.2299, and the average clustering coefficient is 0.0448, as shown in Table 8. In comparison, the SOACN has a significantly smaller average path length and higher clustering coefficient, indicating that the connections between vertexes are relatively close overall. The relationships between vertexes in a small network are very close. It suggests that the SOACN has small-world properties. Hence, risk propagation in the SOACN is fast.

#### 4.5.6. The Scale-Free Property

**Figure 8:**
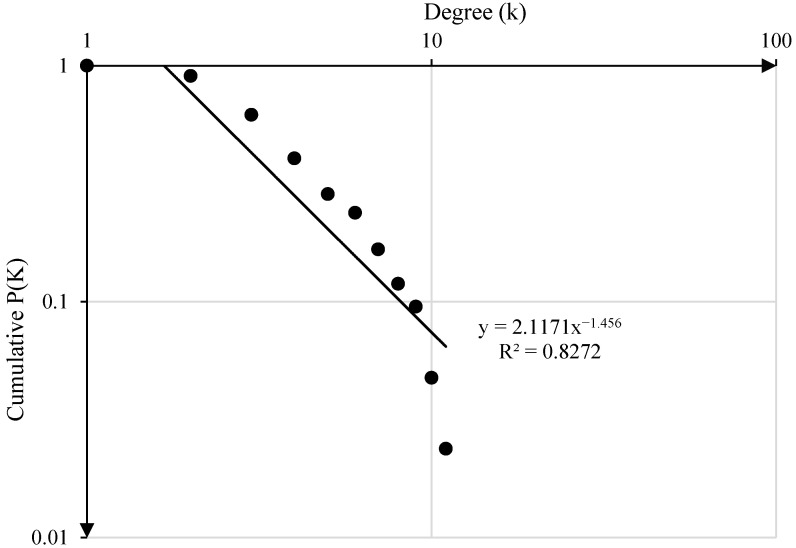
Cumulative distribution of total degree values.

In a scale-free network, the degree value has the characteristics of a power-law function. Figure 8 shows the cumulative degree distribution of all vertexes in the SOACN. The degree-distribution function fits the power-law function P(k) = 2.1171*k^−1.456^, indicating that the SOACN is a scale-free network. The importance of individual vertexes in a scale-free network is relatively higher than other vertexes in the network, which means that a small number of vertexes in the network can affect the structure and function of the network to a greater extent. Therefore, it is necessary to focus on the crucial vertexes in the network and strengthen safety management.

### 4.6. Vulnerability Evaluation of the SOACN 

Subway safety managers can choose various effective measures to reduce or eliminate risks in operation. From the perspective of safety management, the vulnerability of the SOACN is the focus of safety management. Implementing safety precautions in the SOACN and decreasing its connectivity is feasible, which will mitigate safety risks. Therefore, it is necessary to explore how the SOACN is decomposed in the condition of removing an accident or causation vertex, in other words, if a special safety measure is implemented to deal with a particular accident, causative factor or combination of several accidents or causative factors. Network efficiency can reflect the size of the entire network affected when a vertex fails. The vulnerability of the vertexes in the SOACN is judged by the efficiency change ratio of the network after removing each vertex. This index defines the network’s vulnerability as the following equation [14]. In Equation (2), *E*[*G*] represents the network efficiency. Where *D* is a set of interferences, *E*[*D*(*G*,*d*)] signifies the extent of efficiency loss.

(2) $$V(G,D)=\frac{E[G]-E[D(G,d)]}{E[G]}$$

**Figure 9:**
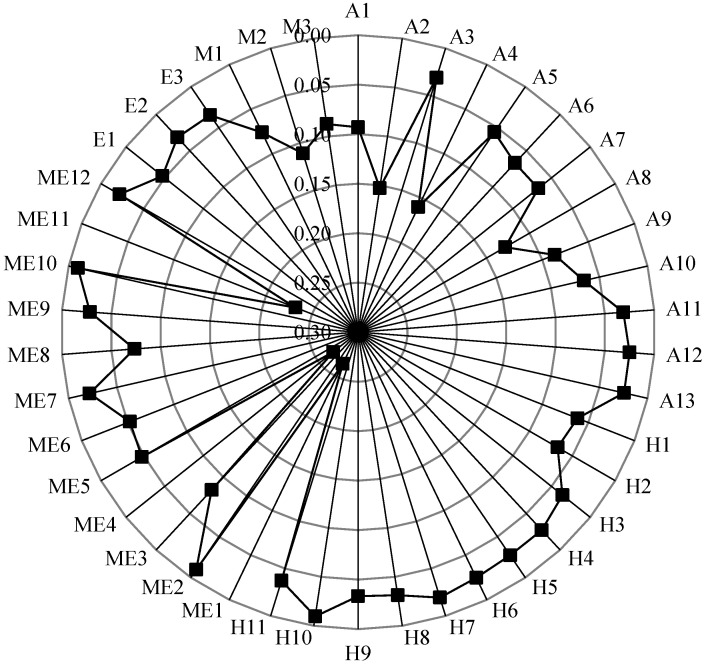
Vulnerability evaluation results.

The original network efficiency of the SOACN is 0.0915. Figure 9 shows the change rate of network efficiency after each vertex is deleted. The more efficiency decreases, the higher the vulnerability increases. It can be seen from the calculation results that among the vertexes of accident types, fire accident (A2) and passenger falling off the rail (A4) are most vulnerable. Safety management should pay attention to preventing these two types of accidents. Among the causation vertexes, improper operation and maintenance of equipment and facilities (ME1), signal failure (ME4) and power failure/power supply interruption/power supply device failure (ME11) are the top three causation vertexes. Compared with other vertexes, they have a considerably more significant influence. All three are machine and equipment factors. Therefore, subway operation safety management personnel should focus on the operation and maintenance of machines and equipment in their daily management to prevent the spread of safety risks from the source.

According to the research results, the topological features and vulnerability of the SOACN can help subway safety managers deepen their understanding of safety risks and their relationships. Critical causative factors that lead to accidents should be considered before reduction or elimination. For instance, a signal system is a core component to ensure the efficient and safe operation of trains. Signal failure (ME4) dramatically influences the regular process of the train. The signal should be used correctly and maintained effectively. Enhancing the maintenance level of the equipment and facilities is critical to subway operation safety [22]. To improve the maintenance of the equipment and facilities, the statistics and analysis of maintenance data provide reliable references to carry out preventive maintenance.

Overall, the vertexes in the SOACN can be divided into three types from the perspective of risk transmission, including the risk-inputted vertex, risk-outputted vertex and intermediary vertex. The characteristics of a vertex need to be considered when making safety-related decisions. In addition, safety measures should target specific risks and the correlation between risks. As shown in Table 4, the average degree in the SOACN is about 4. There are many correlations among safety risks in subway operations. The interconnections require the safety manager to better understand the systems and context behind risks. Analyses that focus on risk interconnections play an essential role in risk response. It is conducive to optimizing safety management to reduce safety risks. However, each subway company has limited safety resources such as staff, equipment, money and material. Optimizing safety-related decisions under resource constraints is a practical problem for safety managers. Furthermore, combining risk control and correlation control simultaneously is also essential.

## 5. Discussion

There are various kinds of safety risks in subway operation, and the characteristics of different safety risks vary greatly, which brings many challenges to safety management. In practice, security resources cannot be evenly distributed. Therefore, safety risks need to be handled differently based on their features. The analysis of the SOACN can identify safety-risk characteristics from multiple perspectives, which is conducive to safety-risk recognition and the development of safety-related measures.

**Figure 10:**
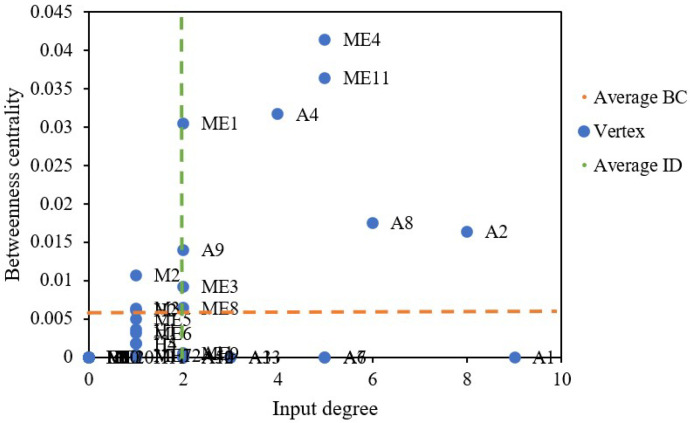
Input degree and betweenness centrality.

Topological characteristics can provide a good reference for safety management. Controlling risk transmission is an essential approach to reducing safety accidents. It identifies the critical features and any associated risks. It helps improve the scientific and rational distribution of safety resources. Betweenness centrality (BC), input degree (ID) and their average values (green and orange dotted lines) are illustrated in Figure 10. signal failure (ME4), power failure/power supply interruption/power supply device failure (ME11), fire accidents (A2), passenger falling off the rail (A4), and train derailment accidents (A8) have high values of BC and ID. This indicates that these two causative factors and three accident types could easily be evoked and transmitted to other risks. 

**Figure 11:**
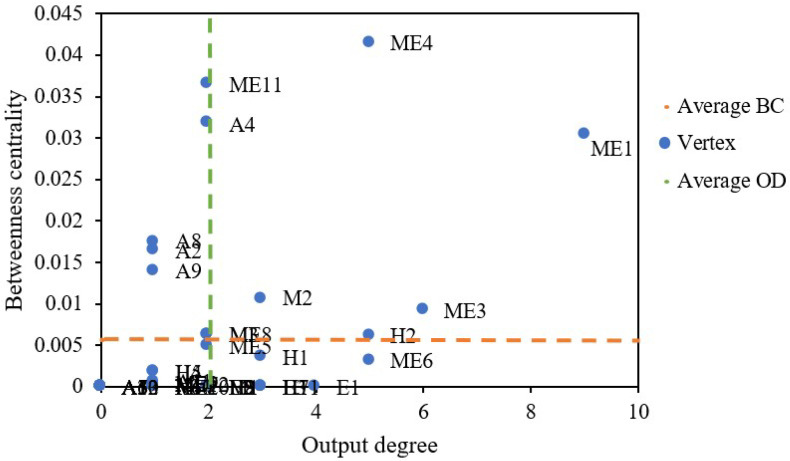
Output degree and betweenness centrality.

Betweenness centrality (BC), output degree (OD) and their average values (green and orange dotted lines) are illustrated in Figure 11. Improper operation and maintenance of equipment and facilities (ME1) and signal failure (ME4) have high values of BC and OD. This indicates that these two causative factors could quickly induce the occurrence of other risks. The onsite safety management personnel should give sufficient focus to these specific safety risks. Applying these results may enhance the management level of risk sources, significantly decreasing the probability of subway operation accidents. 

The potential contributions of this paper can be summarised as follows. Firstly, this paper identifies the propagation path between the accidents and causative factors that lead to subway operation risks and accidents. Mining association rules could discover the potential and indirect correlations in many accident cases and improve knowledge management. Secondly, subway operating companies can develop a targeted management system and strategy to control critical causative factors and eliminate risk chain reactions according to transmission rules. Thirdly, employing targeted emerging technologies for monitoring essential causative factors and risks, such as the internet of things, building information modelling and big data, should be beneficial. The development of new technology and its application in the urban subway industry provide strong support for the digitalization, informatization and intelligent development of subway operation and make smart subway become the hot spot and trend of the industry. Smart subway systems can be constructed to realize the full range of real-time monitoring of personnel, machinery, materials and environment, strengthen safety management and effectively prevent safety accidents.

## 6. Conclusions

Unlike the previous research that explored interconnections among various causative factors, this study employed data mining to integrate accidents and causations in subway operations and built the SOACN. This network model includes the causations, accidents and interrelationships among various accident causations and accidents based on network theory. This study is beneficial to subway safety managers for systematically optimizing safety-related measures to reduce and eliminate safety risks in subway operations.

The topological features of the SOACN and the vulnerability of the SOACN are identified and assessed based on network theory. The degree, betweenness and clustering coefficient of vertexes show apparent discrepancies, and there are some noticeable differences between accidents and causations. The causation vertex set had a lower average degree than the accident vertex set, which signifies that causation vertexes have fewer neighbour vertexes than accident vertexes. This shows that the accident was caused by a combination of factors. The accident vertex set gains a more considerable value of average betweenness centrality than that of human, environmental and management factors, but less than that of the mechanical factor. This means that the mechanical factor has a more significant influence under the control over safety-risk propagation. The neighbour vertexes of an accident vertex are more easily connected than causation vertexes. The fact that accidents do not occur in isolation may explain this phenomenon. To ensure the safe and efficient operation of the subway system, it is essential to prioritize the control of critical accidents and causations, especially the vertexes with high values of both degree and betweenness, such as fire accident (A2), improper operation and maintenance of equipment and facilities (ME1), signal failure (ME4) and power failure/power supply interruption/power supply device failure (ME11). Considering that the safety performance of the equipment may slowly degrade over a long time and eventually lead to severe risks, these results can positively impact early warning to strengthen the maintenance of subway equipment. The value of average path length in the SOACN is 2.4134, indicating that one risk may transmit to another in only two to three steps on average. Preventing the correlation between risks should be implemented to reduce the chain reaction.

Furthermore, topological characteristics were calculated to determine that the SOACN is not only a small-world network but also a scale-free network model. It is demonstrated that the risk propagation in the SOACN is fast, and the SOACN is vulnerable to deliberate attacks. The vulnerability evaluation of the SOACN implied that multiple accidents and causations should not be equally considered due to different roles in the SOACN. Reasonable control of key safety risks is conducive to improving the overall safety level of subway operations. The characteristics of safety risks should be fully considered when making safety-related decisions and formulating safety-related policies. Additionally, it is crucial to provide employees with the necessary training to recognize and respond to safety risks to ensure a safe environment for all passengers and personnel. Safety risks and propagation need to be paid attention to and invested in with security resources.

There are three limitations to this study. Firstly, the weights of vertexes and edges were not assigned when the SOACN was built owing to insufficient data. In practice, it is troublesome to estimate the importance of different kinds of accidents and causative factors accurately and quantitatively. In future studies, the SOACN could be improved in terms of node weights based on a more precise understanding of subway operation safety risks. Secondly, edge failure is not considered in the vulnerability evaluation. In this work, vulnerability assessment believes only a single node provides apparent objects for the safety manager. It is argued that if the safety risks are controlled, the risk of transmission can be substantially reduced. The edge failure could be explored in a future study. Thirdly, the probability of risk occurrence and transmission needs to be explored more deeply. It is suggested that big data analysis based on a large number of case statistics helps study and determine risk probability. Specific control measures for safety risks need to be further developed. In addition, the appropriate measures for making decisions about safety in the condition of safety resource constraints deserve further study.

## Figures and Tables

**Table 1:** Accident type and corresponding code.

Accident Type	Code
Operation delay accident	A1
Fire accident	A2
Explosion accident	A3
Passenger falling onto rails	A4
Train hit people accident	A5
Train collision accident	A6
Train rear-end accident	A7
Train derailment accident	A8
Train door/screen door clamping accident	A9
Poisoning and suffocation accident	A10
Stampede accident	A11
Passenger-falling injury	A12
Station/line flood accident	A13

**Table 2:** Examples of open coding of subway operation accidents’ risk factors.

Accident Case Description	Free Vertex	Categorization
At about 10:00 on 18 February 2003, a passenger in the Joongang subway station in Daegu City, South Korea, sprinkled the flammable material in the plastic can on the seat, set it on fire and ran out of the station, leading to the power failure of the station and the fire ignited the train on the target platform, resulting in 198 deaths and 147 injuries on the two trains.	A passenger sprinkled the flammable material in the plastic can on the seat, set it on fire and ran out of the station	Arson, passengers carrying flammable explosives and other prohibited items
At 9:36 on 5 July 2011, the escalator at Exit A of Zoo Station, Line 4 of the Beijing Subway, had a sliding failure. The ascending escalator suddenly lost control and went downward, causing dozens of subway passengers on the escalator to fall from a height. The accident killed a 13-year-old boy, injured three others seriously and 27 others with a slight injury. The direct cause of the accident happened due to the damage to the fixed parts of the elevator and the displacement of the escalator drive main engine, causing the drive chain to fall off and the escalator to slide down. The Beijing Municipal Bureau of Quality Supervision announced the preliminary investigation results.	The fixed parts of the elevator are damaged, and the escalator drive main engine is displaced, causing the drive chain to fall off and the escalator to slide down	Escalator failure

**Table 3:** Spindle coding of accident risk factors.

Types of Risk Factors	Risk Factors and Their Codes
Human factor (H)	Staff operation error (H1)
	Driver’s illegal operation (H2)
	Passengers forcibly get on and off the train (H3)
	Passengers carrying flammable and explosive and other prohibited items (H4)
	Arson (H5)
	Passengers fight and dispute (H6)
	Terrorist attack (H7)
	Passenger attempted suicide (H8)
	Passenger congestion (H9)
	Improper cigarette butts disposal/smoking (H10)
	Speeding (H11)
Mechanical factor (ME)	Improper operation and maintenance of equipment and facilities (ME1)
	Flammables catch fire in stations and trains (ME2)
	Electrical equipment failure (ME3)
	Signal failure (ME4)
	Escalator failure (ME5)
	Vehicle failure (ME6)
	Line equipment failure (ME7)
	Train door/screen door failure (ME8)
	Catenary failure (ME9)
	Cable short circuit (ME10)
	Power failure/power supply interruption/power supply device failure (ME11)
	Turnout failure (ME12)
Environmental factor (E)	Extreme weather (E1)
	Foreign body invasion limit (E2)
	Construction disturbance near the line (E3)
Management factor (M)	Imperfect safety management system (M1)
	Inadequate safety training (M2)
	Management negligence (M3)

**Table 4:** Strong association rules between accidents and causations.

Serial Number	Consequent	Antecedent	Serial Number	Consequent	Antecedent	Serial Number	Consequent	Antecedent
1	A1	ME4	29	A2	H10	57	A10	H7
2	A1	ME6	30	ME11	ME1	58	A2	H7
3	A1	ME8	31	ME12	ME1	59	ME9	E1
4	ME1	M3	32	A7	ME3	60	ME3	H1
5	A1	ME3	33	A6	ME3	61	A7	H11
6	A1	ME7	34	ME11	E1	62	A8	E2
7	A1	ME11	35	A13	E3	63	A8	H11
8	ME6	ME1	36	A2	ME2	64	H5	H4
9	ME1	M2	37	A4	H6	65	A12	A11
10	A3	H7	38	A7	ME4	66	A11	H9
11	A5	A4	39	A6	ME4	67	A8	ME4
12	ME8	ME1	40	A2	ME6	68	A7	ME6
13	A4	H8	41	A8	ME3	69	A6	ME6
14	ME7	ME1	42	A9	ME8	70	ME5	ME1
15	A2	ME3	43	H4	M3	71	ME4	ME11
16	M3	M1	44	ME11	A4	72	ME4	E1
17	M2	M1	45	A2	A8	73	A3	H8
18	A2	ME10	46	A8	H2	74	ME4	H2
19	H2	M2	47	A4	A9	75	A6	H2
20	A7	H2	48	ME11	E2	76	A11	ME5
21	A12	ME5	49	A4	H3	77	ME4	H1
22	A13	E1	50	ME8	H3	78	A13	H1
23	A1	ME12	51	A2	H5	79	A6	H11
24	A8	ME6	52	A5	ME4	80	A1	E3
25	A10	A2	53	ME4	ME1	81	ME11	E3
26	H1	M2	54	ME9	ME1	82	A9	H9
27	H11	H2	55	ME3	ME1			
28	A1	ME9	56	A3	ME3			

**Table 5:** The average degrees of various types of vertex sets.

Vertex Set	Average Input Degree	Average Output Degree	Average Degree
Accident	4.08	0.46	4.54
Causation	1	2.62	3.62
Causation 1: Human factor	0.45	2.18	2.64
Causation 2: Mechanical factor	1.83	3.00	4.83
Causation 3: Environmental factor	0.00	3.00	3.00
Causation 4: Management factor	0.67	2.33	3.00

**Table 6:** The average betweenness centrality of various types of vertex sets.

Vertex Set	Average Betweenness Centrality
Accident	0.0062
Causation	0.0056
Causation 1: Human factor	0.0012
Causation 2: Mechanical factor	0.0111
Causation 3: Environmental factor	0
Causation 4: Management factor	0.0057

**Table 7:** The average clustering coefficient of various types of vertex sets.

Vertex Set	Average Clustering Coefficient
Accident	0.1178
Causation	0.0433
Causation 1: Human factor	0.0561
Causation 2: Mechanical factor	0.0324
Causation 3: Environmental factor	0.0833
Causation 4: Management factor	0

**Table 8:** Average path length and average clustering coefficient of randomly simulated networks.

Random Network	Average Path Length	Average Clustering Coefficient
1	4.9511	0.0459
2	4.3855	0.0591
3	4.1373	0.0469
4	4.1952	0.0294
5	4.4699	0.0320
6	4.1520	0.0293
7	3.9870	0.0427
8	3.6356	0.0417
9	4.3435	0.0741
10	4.0420	0.0469
Average value	4.2299	0.0448

## Data Availability

All data related to this study is explicitly plotted in the figures in this article.
